# The nutritional composition and impact on UK dietary intakes of meat and plant-based meat alternatives

**DOI:** 10.1038/s41538-025-00577-7

**Published:** 2025-11-03

**Authors:** Mary Gouela, Sokratis Stergiadis, Miriam E. Clegg

**Affiliations:** 1 Department of Animal Sciences, School of Agriculture, Policy and Development, University of Reading, Reading, UK; 2 Department of Food Science and Nutrition, School of the Environment, University of the Aegean, Myrina, Greece; 3 School of Food and Nutritional Sciences, University College Cork, Cork, Ireland

**Keywords:** Nutrition, Risk factors, Agriculture

## Abstract

Meat alternatives are designed to be used as like-for-like replacements for meat; however, meat is a source of key nutrients. Implications for the impact on dietary intakes are unknown. Nutritional information on plant-based meat alternatives (*n* = 475) and meat products (*n* = 754) available in the UK was collected. The products were categorized into food type as per the UK National Diet and Nutrition Survey (NDNS) and into sub-categories: meat (ME), plant-based (PB) and mycoprotein (MP). The NDNS data were used to calculate the intake of meat products across age groups. PB and MP were then substituted for meat intakes, and energy and nutrient intakes were calculated and compared to UK Dietary Reference Values. Price, fat, saturated fat, carbohydrate, sugar, protein, fibre, and energy were different between ME, MP and PB products (*P* < 0.001), leading to changes in nutrient intakes. There was considerable variation between product categories, highlighting the impact of like-for-like replacements on nutrient intakes.

## Introduction

The UK National Food Strategy highlights that for the Government to meet its existing commitments on health, climate and nature, meat production must be reduced by 30% within the decade^1^. Meat production is associated with a significant contribution to the agricultural environmental footprint as livestock production systems use substantial quantities of water and areas of land^2,3^. In the UK, the daily total meat consumption has decreased by 17.4 g over 10 years. (2008/09–2018/19)^4^, and people have become more inclined towards vegan, vegetarian, and plant-based diets. The Food Standards Agency's Food and You 2 survey indicates that the proportion of vegans in England, Wales and Northern Ireland is only between 1% and 2%. However, a survey from 2020 showed that 21% of UK consumers actively reduced the amount of meat in their diet^5^. Furthermore, in 2022, research by Ipsos found that 46% of British people aged 16–75 years were considering reducing their intake of animal products in the future^6^.

As a result, several meat substitutes have made their way into the market, offering alternative options to consuming meat whilst maintaining a similar eating experience, patterns and habits^5^. Meat substitutes are products that are typically processed, most often based on legumes, pulses, fungi, and other plants. Although plant-based meats continue to make up <1% of the world’s meat market, the food industry has accomplished an expansion of the meat substitute market, with the UK experiencing growth from 5% in 2018 to 11% in 2021^7^. In 2022^6^, the Grocer reported that vegan food sales at Aldi were 5 times higher in January 2022 than in January 2021. Aldi also reported a 2.5-times increase in vegan food sales in 2021 compared with 2020^8^ and during 2023, 33% of households in Great Britain bought plant-based meat alternatives at least once^9^. In 2023, the market value of plant-based meat substitutes worldwide was estimated to be worth 10.15 billion in U.S. dollars. This figure is estimated to steadily increase over the coming years and reach roughly 16.78 billion in 2028^10^.

Meat is still a major source of high-quality protein, as well as providing many vitamins and minerals, specifically, vitamin B12, zinc and iron^3^. In the UK, 19% of iron intakes, 31% of zinc intakes for adults 19–64+ years old, 14% of iron intakes, as well as 26% of zinc intakes for children 4–10 years old, come from meat^11^. Many of these essential nutrients cannot be acquired easily in adequate quantities from non-meat sources of food, for example, for adults 19–64+ years old, iron and zinc daily intakes from vegetables and potatoes account for 16% and 11%, respectively^11^. Moreover, for children 4–10 years old, iron and zinc daily intakes from vegetables and potatoes amount to 12% and 10%, respectively^11^. Iron absorption includes haem iron from animal sources and non-haem iron from plant foods. Haem iron, which is found in meats, poultry, and seafood, is absorbed more efficiently and has greater bioavailability compared to non-haem iron^12^. Findings from other studies suggest that individuals following vegetarian and vegan diets may be at higher risk for iron and zinc deficiencies than meat eaters^13,14^. In a Swiss study, the vegan group showed the highest prevalence of inadequate zinc levels, reaching 47%, compared with only 10.8% of omnivores^13^. While iron deficiency can vary significantly between vegans/vegetarians and omnivores, ranging from 66.7% in those following a vegan/vegetarian diet, to 30.8% in those following an omnivorous diet, in a sample of Swedish female high school students, the prevalence of iron deficiency was reported to be 40% among vegan women of reproductive age (iron deficiency criteria <12 ng/mL of ferritin)^15^. This potential risk of nutrient deficiencies might also explain why some consumers are reluctant to follow a vegetarian, vegan or plant-based diet^16^. Nevertheless, meat is also high in saturated fat, and meat consumption significantly increases saturated fat intake, often raising it beyond the recommendations to limit saturated fat intake to less than 10% of total daily dietary energy^11,17,18^. Red and processed meat consumption has also been suggested as an important risk factor for cardiovascular diseases and cancer^3,19,20^.

Plant-based meat alternative (PBMA) products have been launched for many years; as such, the literature on them is growing with regard to their nutrient composition^21–24^. The studies across different countries have provided some basic knowledge around the nutritional quality of meat and PBMA, but some of the studies were limited in terms of samples and nutrient composition, and the implications for nutrient intakes of the population have been assessed in a limited number of studies^24,25^. Exploring the impact on population nutrient intakes is essential given that PBMA are designed to be used as a like-for-like replacement for meat, and the implications for how this impacts dietary intakes are not fully understood. The primary aim of this research was therefore to (i) record the available meat products and PBMA at retail level in the UK (within 5-mile radius from the University of Reading), (ii) create a database on label information regarding their nutrient composition, and cost in order to statistically compare diverse types of plant-based meat alternatives to their corresponding meat products, and (iii) quantify the impact on nutrient intakes from replacement of meat with PBMA for different UK demographics, as described in the NDNS^11^.

## Results

**Fig. 1:**
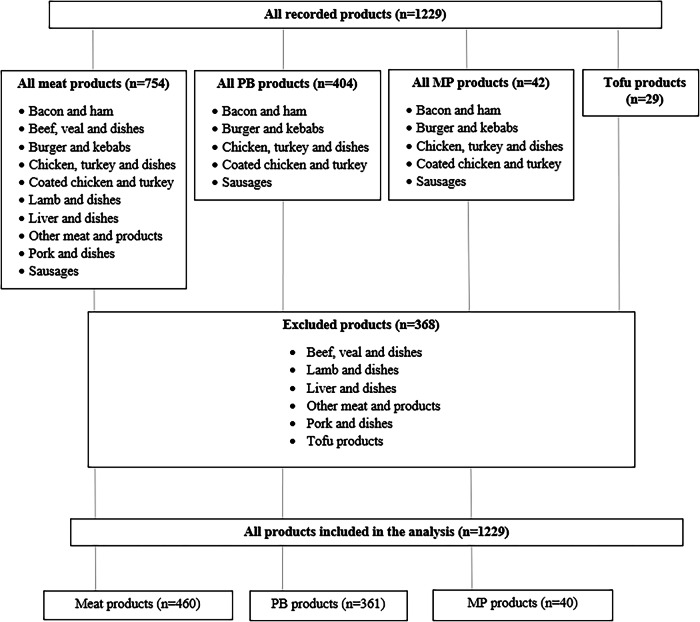
The selection process used for meat, plant-based (PB) and mycoprotein (MP) product inclusion and their classification according to the UK National Diet and Nutrition Survey (NDNS) database.

In total, 475 plant-based meat products and 754 meat products were recorded (Fig. 1).

### Comparison of nutritional composition of meat (ME), plant-based (PB) and mycoprotein (MP) products available on the market

This study initially compared meat (ME), plant-based (PB) and mycoprotein (MP) products’ nutrient composition regarding energy, macronutrients, salt, and price. There was a significant difference in the price, and contents of energy, fat, saturated fat, carbohydrates, sugars, fibre and protein (*P* < 0.001, Table 1) between the three categories of products. When compared with PB and MP, ME products had a lower price (−£5.13/kg and −£3.63/kg, respectively; *P* < 0.001) and lower carbohydrate concentrations (−5.94 and −3.56 g/100 g; *P* < 0.001). They also had higher concentrations of energy (+27 and + 47 kcal/100 g; *P* < 0.001), fat (+4.23 and + 5.20 g/100 g; *P* < 0.001), saturates (+2.58 and + 3.04 g/100 g; *P* < 0.001), and protein (+5.00 and + 6.00 g/100 g; *P* < 0.001). PB products had higher concentrations of sugars than ME and MP (+0.81 and + 0.81 g/100 g, respectively; *P* < 0.001). The concentration of fibre ranged from 0.87 to 5.83 g/100 g, and it differed between ingredients and was highest in MP and lowest in ME (*P* < 0.001).

**Table 1:** Cost and nutrient content of meat, mycoprotein and plant-based products

	Meat			Plant-based			Mycoprotein			
	Mean	SE^1^	*n*^2^	Mean	SE^1^	*n*^2^	Mean	SE^1^	*n*^2^	*P*-values^3^
Price (£/kg)	7.27^b^	±0.199	460	12.4^a^	±0.28	359	10.9^a^	±0.66	40	<0.001
Energy per 100 g (kcal)	229^a^	±2.560	456	202^b^	±3.584	361	182^b^	±8.482	40	<0.001
Fat (g/100 g)	13.7^a^	±0.276	456	9.47^b^	±0.387	361	8.50^b^	±0.915	40	<0.001
Saturates (g/100 g)	4.47^a^	±0.120	456	1.89^b^	±0.168	360	1.43^b^	±0.398	40	<0.001
Carbohydrates (g/100 g)	6.70^b^	±0.30	424	12.64^a^	±0.416	361	10.26^a^	±0.973	40	<0.001
Sugars (g/100 g)	1.40^b^	±0.093	435	2.21^a^	±0.128	361	1.40^b^	±0.303	40	<0.001
Fibre (g/100 g)	0.87^c^	±0.092	395	4.82^b^	±0.128	351	5.83^a^	±0.286	40	<0.001
Protein (g/100 g)	19.3^a^	±0.244	456	14.3^b^	±0.341	361	13.3^b^	±0.807	40	<0.001
Salt (g/100 g)	1.36	±0.032	454	1.44	±0.045	361	1.29	±0.111	39	0.260

^1^SE = standard error of mean.

^2^*n* = number of records.

^3^Significant differences were declared at *P* < 0.05. Different lower-case letters within a row indicate significant differences between product categories (Tukey’s honest significance test; *P* < 0.05).

**Fig. 2:**
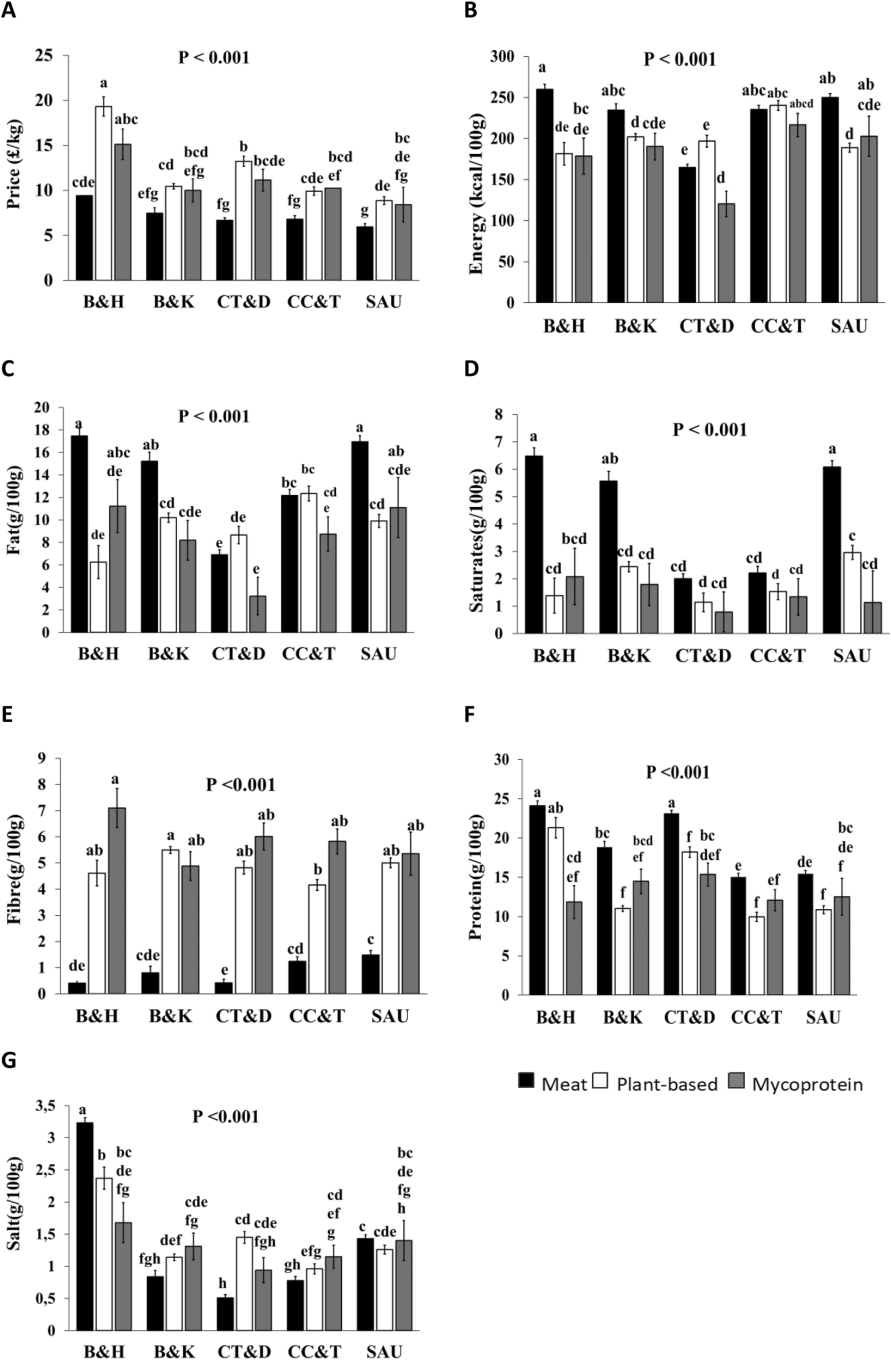
Significant interactions for the effect of main ingredient and type of product on price and contents. Assessment of price **(A)**, energy **(B)**, fat **(C)**, saturates **(D)**, fibre **(E)**, protein **(F)** and salt **(G)**. Main ingredients include; meat, mycoprotein, plant-based alternatives and products include; bacon and ham (B&H); burgers and kebabs, B&K; chicken, turkey and dishes, CT&D; coated chicken and turkey, CC&T; sausages, SAU. Data are mean ± SE, bars labelled with different letters are significantly different (Tukey’s honest significance test; *P* < 0.05).

There was a significant interaction between ingredient and type of product for price, and concentrations of energy, fat, saturated fat, carbohydrate, sugars, fibre, protein and salt (*P* < 0.001, Fig. 2). ME products were significantly cheaper than PB across product types, and cheaper than MP for the chicken turkey and dishes (CT&D) (*P* < 0.001). ME had higher energy content than PB for burger and kebabs (B&K), sausages (SAU), bacon and ham (B&H), and lower energy content for CT&D. The energy content of ME was also higher than MP for B&H, while MP had lower energy content even compared with PB for coated chicken and turkey (CC&T).

ME contained higher amounts of fat and saturated fat than PB for B&H and SAU. For CT&D and CC&T, fat content was similar among all main ingredients (*P* < 0.001, Fig. 2). Saturated fat content in ME was higher than in PB and MP, for B&H, B&K, and SAU. For the rest of the categories, saturated fat content was the same (*P* < 0.001, Fig. 2). Moreover, protein content in ME was higher, than in PB for B&K, CT&D, CC&T, and SAU, whereas protein content in ME was higher, than in MP for B&H (*P* < 0.001, Fig. 2). As far as salt content in ME is concerned, this was higher, than in PB for B&H and CT&D.

Significant differences between ME, MP, PB products in energy, fat, saturated fat, carbohydrates, sugars, protein, fibre and salt content are presented in Tables 2−6. The percentage (%) contribution to nutrient requirements from the consumption of ME, MP and PB for the different demographics, based on their recorded food intakes in the National Diet and Nutrition Survey, is presented in Tables 7−11. Moreover, the % contribution to nutrient requirements from the consumption of all products for the different demographics is presented in Table 12.

**Table 2:** Energy and nutritional content of meat bacon and ham, mycoprotein bacon and ham, and plant-based bacon and ham available on the UK market

Bacon and ham										
Variable	Meat			Plant-based			Mycoprotein			
	Mean	SE	*n*^1^	Mean	SE	*n*^1^	Mean	SE	*n*^1^	*P*-values^2^
Energy (kcal/100 g)	260^a^	±8.9	58	181^b^	±18.9	13	179^b^	±30.4	5	<0.001
Fat (g/100 g)	17.5^a^	±0.97	58	6.25^b^	±2.040	13	11.2^a, b^	±3.29	5	<0.001
Saturated Fat (g/100 g)	6.48^a^	±0.303	58	1.39^b^	±0.713	13	2.08^b^	±1.150	5	<0.001
Carbohydrates (g/100 g)	0.84^c^	±0.227	60	8.28^a^	±0.481	13	3.96^b^	±0.775	5	<0.001
Sugar (g/100 g)	0.52^b^	±0.132	55	2.26^a^	±0.271	13	1.24^a, b^	±0.436	5	<0.001
Fibre (g/100 g)	0.41^c^	±0.172	47	4.58^b^	±0.341	12	7.00^a^	±0.528	5	<0.001
Protein (g/100 g)	24.1^a^	±0.94	58	21.3^a^	±1.98	13	11.8^b^	±3.19	5	<0.001
Salt (g/100 g)	3.23^a^	±0.108	58	2.37^b^	±0.227	13	1.68^b^	±0.410	4	<0.001

^1^SE = standard error of mean.

^2^*n* = number of records.

^3^Significant differences were declared at *P* < 0.05. Different lower-case letters within a row indicate significant differences between product categories (Tukey’s honest significance test; *P* < 0.05).

**Table 3:** Energy and nutritional content of meat burgers and kebabs, mycoprotein burgers and kebabs, and plant-based burgers and kebabs available on the UK market

Burgers and kebabs										
Variable	Meat			Plant-based			Mycoprotein			
	Mean	SE	*n*^1^	Mean	SE	*n*^1^	Mean	SE	*n*^1^	*P*-values^2^
Energy (kcal/100 g)	234^a^	±7.52	42	202^b^	±3.9	156	190^b^	±16.3	9	<0.001
Fat (g/100 g)	15.2^a^	±0.81	42	10.2^b^	±0.420	156	8.19^b^	±1.750	9	<0.001
Saturated fat (g/100 g)	5.57^a^	±0.483	42	2.44^b^	±0.250	156	1.79^b^	±1.040	9	<0.001
Carbohydrates (g/100 g)	5.28^b^	±1.050	42	13.5^a^	±0.547	156	12.2^a^	±2.28	9	<0.001
Sugar (g/100 g)	0.92^b^	±0.268	42	2.27^a^	±0.139	156	1.74^a, b^	±0.578	9	<0.001
Fibre (g/100 g)	0.80^b^	±0.347	41	5.50^a^	±0.742	154	4.88^a^	±0.742	9	<0.001
Protein (g/100 g)	18.8^a^	±0.745	42	11.0^b^	±0.39	156	14.5^b^	±1.61	9	<0.001
Salt (g/100 g)	0.84^b^	±0.060	42	1.14^a^	±0.031	156	1.31^a^	±0.130	9	<0.001

^1^SE = standard error of mean.

^2^*n* = number of records.

^3^Significant differences were declared at *P* < 0.05. Different lower-case letters within a row indicate significant differences between product categories (Tukey’s honest significance test; *P* < 0.05).

**Table 4:** Energy and nutritional content of meat chicken, turkey, and dishes, mycoprotein chicken, turkey and dishes, and plant-based chicken, turkey, and dishes available on the UK market

Chicken, turkey, and dishes										
Variabe	Meat			Plant-based			Mycoprotein			
	Mean	SE	*n*^1^	Mean	SE	*n*^1^	Mean	SE	*n*^1^	*P*-values^2^
Energy (kcal/100 g)	165^b^	±3.2	161	197^a^	±6.1	46	120^c^	±13.0	10	<0.001
Fat (g/100 g)	6.92^a^	±0.369	161	8.67^a^	±0.691	46	3.24^b^	±1.480	10	0.003
Saturated fat (g/100 g)	2.00^a^	±0.109	161	1.14^b^	±0.204	46	0.79^b^	±0.437	10	<0.001
Carbohydrates (g/100 g)	2.74^b^	±0.395	143	9.22^a^	±0.696	46	4.49^b^	±1.490	10	<0.001
Sugar (g/100 g)	0.98^b^	±0.193	143	2.61^a^	±0.340	46	0.84^a, b^	±0.729	10	<0.001
Fibre (g/100 g)	0.42^c^	±0.095	137	4.82^b^	±0.166	45	6.01^a^	±0.351	10	<0.001
Protein (g/100 g)	23.2^a^	±0.37	161	18.2^b^	±0.69	46	15.4^b^	±1.48	10	<0.001
Salt (g/100 g)	0.51^b^	±0.076	161	1.45^a^	±0.143	46	0.94^a, b^	±0.306	10	<0.001

^1^SE = standard error of mean.

^2^*n* = number of records.

^3^Significant differences were declared at *P* < 0.05. Different lower-case letters within a row indicate significant differences between product categories (Tukey’s honest significance test; *P* < 0.05).

**Table 5:** Energy and nutritional content of meat-coated chicken and turkey, mycoprotein-coated chicken and turkey, and plant-based coated chicken and turkey available on the UK market

Coated chicken and turkey										
Variable	Meat			Plant-based			Mycoprotein			
	Mean	SE	*n*^1^	Mean	SE	*n*^1^	Mean	SE	*n*^1^	*P*-values^2^
Energy (kcal/100 g)	235^a^	±3.9	93	240^a^	±4.7	64	216^a^	±10.8	12	0.132
Fat (g/100 g)	12.2^a^	±0.39	93	12.4^a^	±0.47	64	8.73^b^	±1.080	12	0.008
Saturated Fat (g/100 g)	2.21^a^	±0.135	93	1.53^b^	±0.164	63	1.34^a, b^	±0.375	12	0.003
Carbohydrates (g/100 g)	16.0^b^	±0.53	93	19.9^a^	±0.64	64	20.0^a^	±1.48	12	<0.001
Sugar (g/100 g)	1.05^b^	±0.130	92	2.09^a^	±0.156	64	1.42^a, b^	±0.361	12	<0.001
Fibre (g/100 g)	1.24^c^	±0.129	86	4.17^b^	±0.151	63	5.84^a^	±0.346	12	<0.001
Protein (g/100 g)	15.0^a^	±0.38	93	9.95^b^	±0.458	64	12.1^b^	±1.06	12	<0.001
Salt (g/100 g)	0.78^b^	±0.035	93	0.96^a^	±0.042	64	1.15^a^	±0.097	12	<0.001

^1^SE = standard error of mean.

^2^*n* = number of records.

^3^Significant differences were declared at *P* < 0.05. Different lower-case letters within a row indicate significant differences between product categories (Tukey’s honest significance test; *P* < 0.05).

**Table 6:** Energy and nutritional content of meat sausages, mycoprotein sausages, and plant-based sausages available on the UK market

Sausages										
Variable	Meat			Plant-based			Mycoprotein			
	Mean	SE	*n*^1^	Mean	SE	*n*^1^	Mean	SE	*n*^1^	*P*-values^2^
Energy (kcal/100 g)	250^a^	±5.7	102	189^b^	±6.4	82	203^a, b^	±28.9	4	<0.001
Fat (g/100 g)	17.0^a^	±0.61	102	9.90^b^	±0.684	82	11.1^a, b^	±3.10	4	<0.001
Saturated Fat (g/100 g)	6.08^a^	±0.264	102	2.96^b^	±0.294	82	1.12^b^	±1.330	4	<0.001
Carbohydrates (g/100 g)	8.22^b^	±0.600	103	12.4^a^	±0.673	82	10.6^a, b^	±3.05	4	<0.001
Sugar (g/100 g)	1.46^a^	±0.169	103	1.85^a^	±0.190	82	1.75^a^	±0.860	4	0.308
Fibre (g/100 g)	1.48^b^	±0.199	84	5.00^a^	±0.208	77	5.35^a^	±0.912	4	<0.001
Protein (g/100 g)	15.43^a^	±0.409	102	10.9^b^	±0.456	82	12.5^a, b^	±2.06	4	<0.001
Salt (g/100 g)	1.43^a^	±0.038	100	1.26^b^	±0.041	82	1.40^a, b^	±0.189	4	0.013

^1^SE = standard error of mean.

^2^*n* = number of records.

^3^Significant differences were declared at *P* < 0.05. Different lower-case letters within a row indicate significant differences between product categories (Tukey’s honest significance test; *P* < 0.05).

**Table 7:** Per day % energy and nutritional contribution to EAR/DRV/RNI from bacon and ham in a UK population across the lifespan, and change contribution to nutrient intake when substituted for plant-based and mycoprotein alternative products

Variable	Age group	EAR/DRV/RNI requirements	% of RNI Bacon and Ham			*P*-value^1^
			Meat	Plant-based	Mycoprotein	
Energy intake (kcal/d)	Children 1.5–3 years	809	0.92^a^	0.64^b^	0.63^b^	<0.001
	Children 4–10 years	1504	0.91^a^	0.64^b^	0.63^b^	<0.001
	Children 11–18 years	2333	0.89^a^	0.62^b^	0.61^b^	<0.001
	Adults 19–64 years	2395	0.96^a^	0.67^b^	0.66^b^	<0.001
	Adults 65+ years	2097	1.04^a^	0.73^b^	0.72^b^	<0.001
	Adults 65–74 years	2127	1.05^a^	0.73^b^	0.72^b^	<0.001
	Adults 75+ years	2067	1.08^a^	0.76^b^	0.75^b^	<0.001
Fat intake (g/d)	Children 1.5–3 years					
	Children 4–10 years	58.5	1.58^a^	0.56^b^	1.01^a, b^	<0.001
	Children 11–18 years	90.7	1.53^a^	0.55^b^	0.98^a, b^	<0.001
	Adults 19–64 years	93.1	1.66^a^	0.59^b^	1.07^a, b^	<0.001
	Adults 65+ years	81.6	1.81^a^	0.65^b^	1.16^a, b^	<0.001
	Adults 65–74 years	82.7	1.81^a^	0.65^b^	1.16^a, b^	<0.001
	Adults 75+ years	80.4	1.87^a^	0.67^b^	1.20^a, b^	<0.001
Saturated fat Intake (g/d)	Children 1.5–3 years					
	Children 4–10 years	16.7	2.04^a^	0.44^b^	0.66^b^	<0.001
	Children 11–18 years	25.9	1.80^a^	0.42^b^	0.64^b^	<0.001
	Adults 19–64 years	26.6	1.98^a^	0.46^b^	0.69^b^	<0.001
	Adults 65+ years	23.3	2.13^a^	0.50^b^	0.74^b^	<0.001
	Adults 65–74 years	23.6	2.13^a^	0.51^b^	0.76^b^	<0.001
	Adults 75+ years	23.0	2.20^a^	0.52^b^	0.78^b^	<0.001
Carbohydrates Intake (g/d)	Children 1.5–3 years	101	0.03^c^	0.24^a^	0.11^b^	<0.001
	Children 4–10 years	188	0.03^c^	0.23^a^	0.11^b^	<0.001
	Children 11–18 years	292	0.03^c^	0.22^a^	0.11^b^	<0.001
	Adults 19–64 years	299	0.03^c^	0.24^a^	0.12^b^	<0.001
	Adults 65+ years	262	0.03^c^	0.26^a^	0.13^b^	<0.001
	Adults 65–74 years	266	0.03^c^	0.27^a^	0.13^b^	<0.001
	Adults 75+ years	258	0.03^c^	0.27^a^	0.13^b^	<0.001
Sugar intake (g/d)	Children 1.5–3 years	10.1	*	0.59^a^	0.40^a, b^	<0.001
	Children 4–10 years	18.8	*	0.64^a^	0.35^a, b^	<0.001
	Children 11–18 years	29.2	*	0.62^a^	0.34^a, b^	<0.001
	Adults 19–64 years	29.9	*	0.68^a^	0.37^a, b^	<0.001
	Adults 65+ years	26.2	*	0.72^a^	0.47^a, b^	<0.001
	Adults 65–74 years	26.6	*	0.73^a^	0.40^a, b^	<0.001
	Adults 75+ years	25.8	*	0.75^a^	0.43^a, b^	<0.001
Fibre intake (g/d)	Children 1.5–3 years	15.0	0.07^c^	0.87^b^	1.33^a^	<0.001
	Children 4–10 years	20.0	0.10^c^	1.22^b^	1.88^a^	<0.001
	Children 11–18 years	25.0	0.12^c^	1.47^b^	2.27^a^	<0.001
	Adults 19–64 years	30.0	0.12^c^	1.37^b^	2.10^a^	<0.001
	Adults 65+ years	30.0	0.11^c^	1.30^b^	2.00^a^	<0.001
	Adults 65–74 years	30.0	0.12^c^	1.31^b^	2.03^a^	<0.001
	Adults 75+ years	30.0	0.12^c^	1.32^b^	2.04^a^	<0.001
Protein intake (g/d)	Children 1.5–3 years	14.5	4.76^a^	4.21^a^	2.34^b^	<0.001
	Children 4–10 years	24.0	5.33^a^	4.71^a^	2.63^b^	<0.001
	Children 11–18 years	45.8	4.18^a^	3.70^a^	2.05^b^	<0.001
	Adults 19–64 years	50.3	4.26^a^	3.76^a^	2.09^b^	<0.001
	Adults 65+ years	73.7	2.76^a^	2.44^a^	1.35^b^	<0.001
	Adults 65–74 years	73.7	2.90^a^	2.48^a^	1.38^b^	<0.001
	Adults 75+ years	73.7	2.82^a^	2.49^a^	1.38^b^	<0.001
Salt intake (g/d)	Children 1.5–3 years	2.00	4.50^a^	3.50^b^	2.50^b^	<0.001
	Children 4–10 years	4.00	4.25^a^	3.17^b^	2.17^b^	<0.001
	Children 11–18 years	6.00	4.28^a^	3.17^b^	2.17^b^	<0.001
	Adults 19–64 years	6.00	4.78^a^	3.50^b^	2.50^b^	<0.001
	Adults 65+ years	6.00	4.50^a^	3.39^b^	2.39^b^	<0.001
	Adults 65–74 years	6.00	4.67^a^	3.39^b^	2.39^b^	<0.001
	Adults 75+ years	6.00	4.67^a^	3.39^b^	2.39^b^	<0.001

^1^ Significant differences were declared at *P* < 0.05. Different lower-case letters within a row indicate significant differences between product categories (Tukey’s honest significance test; *P* < 0.05).

**Table 8:** Per day % energy and nutritional contribution to EAR/DRV/RNI from burgers and kebabs in a UK population across the lifespan, and change contribution to nutrient intake when substituted for plant-based and mycoprotein alternative products

Variable	Age group	EAR/DRV/RNI requirements	% of RNI Burgers and Kebabs			*P*-value^1^
			Meat	Plant-based	Mycoprotein	
Energy intake (kcal/d)	Children 1.5–3 years	809	0.18^a^	0.15^b^	0.14^b^	<0.001
	Children 4–10 years	1504	0.55^a^	0.47^b^	0.45^b^	<0.001
	Children 11–18 years	2333	0.81^a^	0.70^b^	0.66^b^	<0.001
	Adults 19–64 years	2395	0.64^a^	0.55^b^	0.52^b^	<0.001
	Adults 65+ years	2097	0.14^a^	0.12^b^	0.12^b^	<0.001
	Adults 65–74 years	2127	0.14^a^	0.12^b^	0.12^b^	<0.001
	Adults 75+ years	2067	0.15^a^	0.09^b^	0.08^b^	<0.001
Fat intake (g/d)	Children 1.5–3 years					
	Children 4–10 years	58.5	0.92^a^	0.62^b^	0.50^b^	<0.001
	Children 11–18 years	90.7	1.36^a^	0.91^b^	0.73^b^	<0.001
	Adults 19–64 years	93.1	1.07^a^	0.72^b^	0.58^b^	<0.001
	Adults 65+ years	81.6	0.24^a^	0.16^b^	0.13^b^	<0.001
	Adults 65–74 years	82.7	0.24^a^	0.16^b^	0.13^b^	<0.001
	Adults 75+ years	80.4	0.17^a^	0.11^b^	0.09^b^	<0.001
Saturated fat Intake (g/d)	Children 1.5–3 years					
	Children 4–10 years	16.7	1.18^a^	0.52^b^	0.38^b^	<0.001
	Children 11–18 years	25.9	1.74^a^	0.76^b^	0.55^b^	<0.001
	Adults 19–64 years	26.6	1.38^a^	0.60^b^	0.44^b^	<0.001
	Adults 65+ years	23.3	0.30^a^	0.13^b^	0.10^b^	<0.001
	Adults 65–74 years	23.6	0.30^a^	0.14^b^	0.10^b^	<0.001
	Adults 75+ years	23.0	0.23^a^	0.09^b^	0.07^b^	<0.001
Carbohydrates intake (g/d)	Children 1.5–3 years	101	0.03^b^	0.08^a^	0.07^a^	<0.001
	Children 4–10 years	188	0.10^b^	0.25^a^	0.23^a^	<0.001
	Children 11–18 years	291	0.15^b^	0.37^a^	0.34^a^	<0.001
	Adults 19–64 years	299	0.12^b^	0.29^a^	0.27^a^	<0.001
	Adults 65+ years	262	0.03^b^	0.06^a^	0.06^a^	<0.001
	Adults 65–74 years	266	0.03^b^	0.07^a^	0.06^a^	<0.001
	Adults 75+ years	258	0.02^b^	0.05^a^	0.04^a^	<0.001
Sugar intake (g/d)	Children 1.5–3 years	10.1	*	0.10^a^	0.10^a, b^	<0.001
	Children 4–10 years	18.8	*	0.41^a^	0.34^a, b^	<0.001
	Children 11–18 years	29.2	*	0.63^a^	0.48^a, b^	<0.001
	Adults 19–64 years	29.9	*	0.50^a^	0.38^a, b^	<0.001
	Adults 65+ years	26.2	*	0.11^a^	0.08^a, b^	<0.001
	Adults 65–74 years	26.6	*	0.11^a^	0.09^a, b^	<0.001
	Adults 75+ years	25.8	*	0.08^a^	0.06^a, b^	<0.001
Fibre intake (g/d)	Children 1.5–3 years	15.0	0.00^b^	0.20^a^	0.20^a^	<0.001
	Children 4–10 years	20.0	0.15^b^	0.97^a^	0.85^a^	<0.001
	Children 11–18 years	25.0	0.25^b^	1.79^a^	1.59^a^	<0.001
	Adults 19–64 years	30.0	0.18^b^	1.20^a^	1.07^a^	<0.001
	Adults 65+ years	30.0	0.03^b^	0.23^a^	0.21^a^	<0.001
	Adults 65–74 years	30.0	0.03^b^	0.23^a^	0.21^a^	<0.001
	Adults 75+ years	30.0	0.03^b^	0.16^a^	0.14^a^	<0.001
Protein intake (g/d)	Children 1.5–3 years	14.5	0.76^a^	0.48^b^	0.62^b^	<0.001
	Children 4–10 years	24.0	2.75^a^	1.61^b^	2.13^b^	<0.001
	Children 11–18 years	45.9	3.32^a^	1.94^b^	2.55^b^	<0.001
	Adults 19–64 years	50.3	2.46^a^	1.43^b^	1.89^b^	<0.001
	Adults 65+ years	73.7	0.33^a^	0.19^b^	0.25^b^	<0.001
	Adults 65–74 years	73.7	0.33^a^	0.19^b^	0.26^b^	<0.001
	Adults 75+ years	73.7	0.23^a^	0.14^b^	0.18^b^	<0.001
Salt intake (g/d)	Children 1.5–3 years	2.00	0.50^b^	0.50^a^	0.50^a^	<0.001
	Children 4–10 years	4.00	0.75^b^	1.00^a^	1.17^a^	<0.001
	Children 11–18 years	6.00	1.06^b^	1.56^a^	1.78^a^	<0.001
	Adults 19–64 years	6.00	0.94^b^	1.22^a^	1.50^a^	<0.001
	Adults 65+ years	6.00	0.17^b^	0.22^a^	0.28^a^	<0.001
	Adults 65–74 years	6.00	0.17^b^	0.22^a^	0.33^a^	<0.001
	Adults 75+ years	6.00	0.17^b^	0.17^a^	0.17^a^	<0.001

^1^Significant differences were declared at *P* < 0.05. Different lower-case letters within a row indicate significant differences between product categories (Tukey’s honest significance test; *P* < 0.05).

**Table 9:** Per day % energy and nutritional contribution to EAR/DRV/RNI from chicken, turkey and dishes in a UK population across the lifespan and change contribution to nutrient intake when substituted for plant-based and mycoprotein alternative products

Variable	Age group	EAR/DRV/RNI requirements	% of RNI chicken, turkey and dishes			*P*-value^1^
			Meat	Plant-based	Mycoprotein	
Energy Intake (kcal/d)	Children 1.5–3 years	809	3.44^b^	4.11^a^	2.51^c^	<0.001
	Children 4–10 years	1504	2.87^b^	3.42^a^	2.09^c^	<0.001
	Children 11–18 years	2333	3.29^b^	3.93^a^	2.40^c^	<0.001
	Adults 19–64 years	2395	4.04^b^	4.83^a^	2.95^c^	<0.001
	Adults 65+ years	2097	3.18^b^	3.80^a^	3.37^c^	<0.001
	Adults 65–74 years	2127	3.19^b^	3.81^a^	2.29^c^	<0.001
	Adults 75+ years	2067	2.97^b^	3.54^a^	2.40^c^	<0.001
Fat intake (g/d)	Children 1.5–3 years					
	Children 4–10 years	58.5	3.09^a^	3.88^a^	1.45^b^	0.003
	Children 11–18 years	90.7	3.56^a^	4.45^a^	1.66^b^	0.003
	Adults 19–64 years	93.1	4.37^a^	5.48^a^	2.04^b^	0.003
	Adults 65+ years	81.6	3.44^a^	4.30^a^	1.61^b^	0.003
	Adults 65–74 years	82.7	3.45^a^	4.32^a^	1.61^b^	0.003
	Adults 75+ years	80.4	3.20^a^	4.01^a^	1.51^b^	0.003
Saturated fat intake (g/d)	Children 1.5–3 years					
	Children 4–10 years	16.7	3.13^a^	1.80^b^	1.24^b^	<0.001
	Children 11–18 years	25.9	3.59^a^	2.06^b^	1.41^b^	<0.001
	Adults 19–64 years	26.6	4.42^a^	2.52^b^	1.74^b^	<0.001
	Adults 65+ years	23.3	3.46^a^	1.97^b^	1.39^b^	<0.001
	Adults 65–74 years	23.6	3.48^a^	2.00^b^	1.37^b^	<0.001
	Adults 75+ years	23.0	3.25^a^	1.86^b^	1.28^b^	<0.001
Carbohydrates intake (g/d)	Children 1.5–3 years	101	0.49^b^	1.54^a^	0.75^b^	<0.001
	Children 4–10 years	188	0.41^b^	1.28^a^	0.63^b^	<0.001
	Children 11–18 years	292	0.47^b^	1.47^a^	0.72^b^	<0.001
	Adults 19–64 years	299	0.58^b^	1.81^a^	0.88^b^	<0.001
	Adults 65+ years	262	0.46^b^	1.42^a^	0.69^b^	<0.001
	Adults 65–74 years	266	0.46^b^	1.43^a^	0.70^b^	<0.001
	Adults 75+ years	258	0.43^b^	1.33^a^	0.65^b^	<0.001
Sugar intake (g/d)	Children 1.5–3 years	10.1	*	4.35^a^	1.38^a, b^	<0.001
	Children 4–10 years	18.8	*	3.62^a^	1.17^a, b^	<0.001
	Children 11–18 years	29.2	*	4.18^a^	1.35^a, b^	<0.001
	Adults 19–64 years	29.9	*	5.12^a^	1.64^a, b^	<0.001
	Adults 65+ years	26.2	*	4.04^a^	1.30^a, b^	<0.001
	Adults 65–74 years	26.6	*	4.04^a^	1.30^a, b^	<0.001
	Adults 75+ years	25.8	*	3.75^a^	1.20 ^a, b^	<0.001
Fibre intake (g/d)	Children 1.5–3 years	15.00	0.47^c^	5.40^b^	6.80^a^	<0.001
	Children 4–10 years	20.00	0.55^c^	6.30^b^	7.85^a^	<0.001
	Children 11–18 years	25.00	0.79^c^	8.99^b^	11.2^a^	<0.001
	Adults 19–64 years	30.00	0.83^c^	9.44^b^	11.8^a^	<0.001
	Adults 65+ years	30.00	0.57^c^	6.51^b^	8.12^a^	<0.001
	Adults 65–74 years	30.00	0.57^c^	6.62^b^	8.27^a^	<0.001
	Adults 75+ years	30.00	0.51^c^	5.98^b^	7.46^a^	<0.001
Protein intake (g/d)	Children 1.5–3 years	14.5	27.0^a^	21.2^b^	17.9^b^	<0.001
	Children 4–10 years	24.0	25.2^a^	19.8^b^	16.7^b^	<0.001
	Children 11–18 years	45.9	23.5^a^	18.5^b^	15.6^b^	<0.001
	Adults 19–64 years	50.6	27.1^a^	21.3^b^	18.0^b^	<0.001
	Adults 65+ years	73.7	12.7^a^	10.0^b^	8.44^b^	<0.001
	Adults 65–74 years	73.7	13.0^a^	10.2^b^	8.58^b^	<0.001
	Adults 75+ years	73.7	11.7^a^	9.19^b^	7.75^b^	<0.001
Salt intake (g/d)	Children 1.5–3 years	2.00	4.50^b^	12.5^a^	8.00^a, b^	<0.001
	Children 4–10 years	4.00	3.33^b^	9.50^a^	6.17^a, b^	<0.001
	Children 11–18 years	6.00	3.94^b^	11.3^a^	7.33^a, b^	<0.001
	Adults 19–64 years	6.00	5.00^b^	14.2^a^	9.22^a, b^	<0.001
	Adults 65+ years	6.00	3.44^b^	9.78^a^	6.33^a, b^	<0.001
	Adults 65–74 years	6.00	3.56^b^	10.0^a^	6.44^a, b^	<0.001
	Adults 75+ years	6.00	3.17^b^	8.94^a^	5.83^a, b^	<0.001

^1^Significant differences were declared at *P* < 0.05. Different lower-case letters within a row indicate significant differences between product categories (Tukey’s honest significance test; *P* < 0.05).

**Table 10:** Per day % energy and nutritional contribution to EAR/DRV/RNI from coated chicken and turkey in a UK population across the lifespan and change contribution to nutrient intake when substituted for plant-based and mycoprotein alternative products

Variable	Age group	EAR/DRV/RNI requirements	% of RNI coated chicken and turkey			*P*-value^1^
			Meat	Plant-based	Mycoprotein	
Energy intake (kcal/d)	Children 1.5–3 years	809	2.62^a^	2.67^a^	2.41^a^	0.132
	Children 4–10 years	1504	1.98^a^	2.02^a^	1.82^a^	0.132
	Children 11–18 years	2333	1.69^a^	1.72^a^	1.55^a^	0.132
	Adults 19–64 years	2395	0.71^a^	0.73^a^	0.66^a^	0.132
	Adults 65+ years	2097	0.32^a^	0.32^a^	0.29^a^	0.132
	Adults 65–74 years	2127	0.32^a^	0.32^a^	0.29^a^	0.132
	Adults 75+ years	2067	0.19^a^	0.19^a^	0.18^a^	0.132
Fat intake (g/d)	Children 1.5–3 years					
	Children 4–10 years	58.5	2.63^a^	2.67^a^	1.89^b^	0.008
	Children 11–18 years	90.7	2.25^a^	2.28^a^	1.61^b^	0.008
	Adults 19–64 years	93.1	0.95^a^	0.96^a^	0.68^b^	0.008
	Adults 65+ years	81.6	0.42^a^	0.43^a^	0.30^b^	0.008
	Adults 65–74 years	82.7	0.42^a^	0.43^a^	0.30^b^	0.008
	Adults 75+ years	80.4	0.25^a^	0.26^a^	0.18^b^	0.008
Saturated fat intake (g/d)	Children 1.5–3 years					
	Children 4–10 years	16.7	1.68^a^	1.16^b^	1.02^a, b^	0.003
	Children 11–18 years	25.9	1.41^a^	0.99^b^	0.86^a, b^	0.003
	Adults 19–64 years	26.6	0.60^a^	0.41^b^	0.38^a, b^	0.003
	Adults 65+ years	23.3	0.27^a^	0.19^b^	0.16^a, b^	0.003
	Adults 65–74 years	23.6	0.27^a^	0.18^b^	0.16^a, b^	0.003
	Adults 75+ years	23.0	0.16^a^	0.12^b^	0.09^a, b^	0.003
Carbohydrates intake (g/d)	Children 1.5–3 years	101	1.42^b^	1.77^a^	1.78^a^	<0.001
	Children 4–10 years	188	1.08^b^	1.34^a^	1.34^a^	<0.001
	Children 11–18 years	292	0.92^b^	1.14^a^	1.15^a^	<0.001
	Adults 19–64 years	299	0.39^b^	0.48^a^	0.48^a^	<0.001
	Adults 65+ years	262	0.17^b^	0.21^a^	0.21^a^	<0.001
	Adults 65–74 years	266	0.17^b^	0.22^a^	0.22^a^	<0.001
	Adults 75+ years	258	0.10^b^	0.13^a^	0.13^a^	<0.001
Sugar intake (g/d)	Children 1.5–3 years	10.1	*	1.88^a^	1.29^a, b^	<0.001
	Children 4–10 years	18.8	*	1.40^a^	0.96^a, b^	<0.001
	Children 11–18 years	29.2	*	1.20^a^	0.81^a, b^	<0.001
	Adults 19–64 years	29.9	*	0.50^a^	0.35^a, b^	<0.001
	Adults 65+ years	26.2	*	0.23^a^	0.15^a, b^	<0.001
	Adults 65-74 years	26.6	*	0.23^a^	0.16^a, b^	<0.001
	Adults 75+ years	25.8	*	0.13^a^	0.09^a, b^	<0.001
Fibre Intake (g/d)	Children 1.5–3 years	15.0	0.73^c^	2.47^b^	3.47^a^	<0.001
	Children 4–10 years	20.0	0.80^c^	2.63^b^	3.68^a^	<0.001
	Children 11–18 years	25.0	0.83^c^	2.79^b^	3.89^a^	<0.001
	Adults 19–64 years	30.0	0.30^c^	1.01^b^	1.40^a^	<0.001
	Adults 65+ years	30.0	0.11^c^	0.40^b^	0.54^a^	<0.001
	Adults 65–74 years	30.0	0.12^c^	0.40^b^	0.57^a^	<0.001
	Adults 75+ years	30.0	0.07^c^	0.23^b^	0.33^a^	<0.001
Protein intake (g/d)	Children 1.5–3 years	14.5	9.38^a^	6.21^b^	7.52^b^	<0.001
	Children 4–10 years	24.0	7.92^a^	5.25^b^	6.36^b^	<0.001
	Children 11–18 years	45.9	5.49^a^	3.63^b^	4.40^b^	<0.001
	Adults 19–64 years	50.3	2.18^a^	1.43^b^	1.74^b^	<0.001
	Adults 65+ years	73.7	0.57^a^	0.38^b^	0.46^b^	<0.001
	Adults 65–74 years	73.7	0.58^a^	0.38^b^	0.47^b^	<0.001
	Adults 75+ years	73.7	0.34^a^	0.23^b^	0.27^b^	<0.001
Salt intake (g/d)	Children 1.5–3 years	2.00	3.50^b^	4.50^a^	5.00^a^	<0.001
	Children 4–10 years	4.00	2.42^b^	3.00^a^	3.67^a^	<0.001
	Children 11–18 years	6.00	2.17^b^	2.67^a^	3.17^a^	<0.001
	Adults 19–64 years	6.00	0.94^b^	1.17^a^	1.33^a^	<0.001
	Adults 65+ years	6.00	0.33^b^	0.44^a^	0.56^a^	<0.001
	Adults 65–74 years	6.00	0.39^b^	0.44^a^	0.56^a^	<0.001
	Adults 75+ years	6.00	0.17^b^	0.28^a^	0.33^a^	<0.001

^1^Significant differences were declared at *P* < 0.05. Different lower-case letters within a row indicate significant differences between product categories (Tukey’s honest significance test; *P* < 0.05).

**Table 11:** Per day % energy and nutritional contribution to EAR/DRV/RNI from sausages in a UK population across the lifespan and change contribution to nutrient intake when substituted for plant-based and mycoprotein alternative products

Variable	Age group	EAR/DRV/RNI Requirements	% of RNI sausages			*P*-value^1^
			Meat	Plant-based	Mycoprotein	
Energy intake (kcal/d)	Children 1.5–3 years	809	2.37^a^	1.79^b^	1.92^a, b^	<0.001
	Children 4–10 years	1504	2.19^a^	1.66^b^	1.78^a, b^	<0.001
	Children 11–18 years	2333	1.29^a^	0.98^b^	1.05^a, b^	<0.001
	Adults 19–64 years	2395	1.10^a^	0.83^b^	0.89^a, b^	<0.001
	Adults 65+ years	2097	0.89^a^	0.68^b^	0.73^a, b^	<0.001
	Adults 65–74 years	2127	0.90^a^	0.68^b^	0.73^a, b^	<0.001
	Adults 75+ years	2067	0.70^a^	0.53^b^	0.57^a, b^	<0.001
Fat intake (g/d)	Children 1.5–3 years					
	Children 4–10 years	58.5	3.83^a^	2.24^b^	2.51^a, b^	<0.001
	Children 11–18 years	90.7	2.26^a^	1.32^b^	1.48^a, b^	<0.001
	Adults 19–64 years	93.1	1.91^a^	1.12^b^	1.25^a, b^	<0.001
	Adults 65+ years	81.6	1.57^a^	0.92^b^	1.02^a, b^	<0.001
	Adults 65–74 years	82.7	1.57^a^	0.91^b^	1.03^a, b^	<0.001
	Adults 75+ years	80.4	1.22^a^	0.71^b^	0.80^a, b^	<0.001
Saturated fat intake (g/d)	Children 1.5–3 years					
	Children 4–10 years	16.7	4.79^a^	2.35^b^	0.90^b^	<0.001
	Children 11–18 years	25.9	2.84^a^	1.38^b^	0.54^b^	<0.001
	Adults 19–64 years	26.1	2.41^a^	1.17^b^	0.44^b^	<0.001
	Adults 65+ years	23.3	1.96^a^	0.96^b^	0.36^b^	<0.001
	Adults 65–74 years	23.6	1.96^a^	0.96 ^b^	0.38^b^	<0.001
	Adults 75+ years	23.0	1.54^a^	0.75^b^	0.28^b^	<0.001
Carbohydrates intake (g/d)	Children 1.5–3 years	101	0.63^b^	0.94^a^	0.80^a, b^	<0.001
	Children 4–10 years	188	0.58^b^	0.87^a^	0.74^a, b^	<0.001
	Children 11–18 years	292	0.34^b^	0.51^a^	0.44^a, b^	<0.001
	Adults 19–64 years	299	0.29^b^	0.43^a^	0.37^a, b^	<0.001
	Adults 65+ years	262	0.24^b^	0.36^a^	0.30^a, b^	<0.001
	Adults 65–74 years	266	0.24^b^	0.36^a^	0.30^a, b^	<0.001
	Adults 75+ years	258	0.19^b^	0.28^a^	0.24^a, b^	<0.001
Sugar intake (g/d)	Children 1.5–3 years	10.1	*	1.38^a^	1.29^a^	0.308
	Children 4–10 years	18.8	*	1.29^a^	1.24^a^	0.308
	Children 11–18 years	29.2	*	0.77^a^	0.73^a^	0.308
	Adults 19–64 years	29.9	*	0.65^a^	0.61^a^	0.308
	Adults 65+ years	26.2	*	0.52^a^	0.51^a^	0.308
	Adults 65–74 years	26.6	*	0.54^a^	0.50^a^	0.308
	Adults 75+ years	25.8	*	0.43^a^	0.39^a^	0.308
Fibre intake (g/d)	Children 1.5–3 years	15.0	0.73^b^	2.53^a^	2.73^a^	<0.001
	Children 4–10 years	20.0	0.98^b^	3.30^a^	3.53^a^	<0.001
	Children 11–18 years	25.0	0.72^b^	2.40^a^	2.59^a^	<0.001
	Adults 19–64 years	30.0	0.52^b^	1.76^a^	1.87^a^	<0.001
	Adults 65+ years	30.0	0.37^b^	1.26^a^	1.34^a^	<0.001
	Adults 65–74 years	30.0	0.38^b^	1.28^a^	1.37^a^	<0.001
	Adults 75+ years	30.0	0.29^b^	0.97^a^	1.03^a^	<0.001
Protein intake (g/d)	Children 1.5–3 years	14.5	8.14^a^	5.72^b^	6.62^a, b^	<0.001
	Children 4–10 years	24.0	8.49^a^	5.97^b^	6.88^a, b^	<0.001
	Children 11–18 years	45.9	4.07^a^	2.86^b^	3.29^a, b^	<0.001
	Adults 19–64 years	50.3	3.22^a^	2.27^b^	2.61^a, b^	<0.001
	Adults 65+ years	73.7	1.57^a^	1.11^b^	1.27^a, b^	<0.001
	Adults 65–74 years	73.7	1.60^a^	1.13^b^	1.30^a, b^	<0.001
	Adults 75+ years	73.7	1.21^a^	0.85^b^	0.99^a, b^	<0.001
Salt intake (g/d)	Children 1.5–3 years	2.00	5.50^a^	5.00^b^	5.50^a, b^	0.013
	Children 4–10 years	4.00	4.75^a^	4.25^b^	4.67^a, b^	0.013
	Children 11–18 years	6.00	2.83^a^	2.50^b^	2.83^a, b^	0.013
	Adults 19–64 years	6.00	2.50^a^	2.22^b^	2.50^a, b^	0.013
	Adults 65+ years	6.00	1.83^a^	1.56^b^	1.72^a, b^	0.013
	Adults 65–74 years	6.00	1.83^a^	1.67^b^	1.78^a, b^	0.013
	Adults 75+ years	6.00	1.39^a^	1.22^b^	1.33^a, b^	0.013

^1^Significant differences were declared at *P* < 0.05. Different lower-case letters within a row indicate significant differences between product categories (Tukey’s honest significance test; *P* < 0.05).

**Table 12:** Per day % energy and nutritional contribution to EAR/DRV/RNI from all meat in a UK population across the lifespan and change in contribution to nutrient intake when substituted for plant-based and mycoprotein alternative products

Variable	Age group	EAR/DRV/RNI requirements	% RNI of all products			*P*-value^1^
			Meat	Plant-based	Mycoprotein	
Energy intake (kcal/d)	Children 1.5–3 years	809	9.52^a^	9.40^b^	7.60^c^	<0.000
	Children 4–10 years	1504	8.50^a^	8.20^b^	6.80^c^	<0.000
	Children 11–18 years	2333	7.97^a^	7.90^b^	6.30^c^	<0.000
	Adults 19–64 years	2395	7.46^a^	7.60^b^	5.70^c^	<0.000
	Adults 65+ years	2097	5.58^a^	5.70^b^	4.20^c^	<0.000
	Adults 65–74 years	2127	5.92^a^	5.70^b^	4.20^c^	<0.000
	Adults 75+ years	2067	5.04^a^	5.10^b^	3.70^c^	<0.000
Fat intake (g/d)	Children 1.5–3 years					
	Children 4–10 years	58.5	12.06^a^	9.97^b^	7.35^b^	<0.000
	Children 11–18 years	90.7	10.94^a^	9.51^b^	6.47^b^	<0.000
	Adults 19–64 years	93.1	9.97^a^	8.87^b^	5.63^b^	<0.000
	Adults 65+ years	81.6	7.46^a^	6.45^b^	4.22^b^	<0.000
	Adults 65–74 years	82.7	7.49^a^	6.47^b^	4.24^b^	<0.000
	Adults 75+ years	80.4	6.72^a^	5.77^b^	3.78^b^	<0.000
Saturated fat intake (g/d)	Children 1.5–3 years					
	Children 4–10 years	16.7	11.67^a^	5.67^b^	3.80^b^	<0.000
	Children 11–18 years	25.9	10.53^a^	5.09^b^	3.64^b^	<0.000
	Adults 19–64 years	26.6	9.97^a^	4.70^b^	3.36^b^	<0.000
	Adults 65+ years	23.3	7.59^a^	3.41^b^	2.50^b^	<0.000
	Adults 65–74 years	23.6	7.62^a^	3.43^b^	2.51^b^	<0.000
	Adults 75+ years	23.0	6.90^a^	3.02^b^	2.29^b^	<0.000
Carbohydrates intake (g/d)	Children 1.5–3 years	101	2.61^b^	4.6^a^	3.5^a^	<0.000
	Children 4–10 years	188	2.20^b^	4.0^a^	3.1^a^	<0.000
	Children 11–18 years	292	1.91^b^	3.7^a^	2.8^a^	<0.000
	Adults 19–64 years	299	1.40^b^	3.3^a^	2.1^a^	<0.000
	Adults 65+ years	262	0.92^b^	2.3^a^	1.4^a^	<0.000
	Adults 65–74 years	266	0.93^b^	2.3^a^	1.4^a^	<0.000
	Adults 75+ years	258	0.77^b^	2.1^a^	1.2^a^	<0.000
Sugar intake (g/d)	Children 1.5–3 years	10.1	*	8.40^a^	4.45^b^	<0.000
	Children 4–10 years	18.8	*	7.40^a^	4.03^b^	<0.000
	Children 11–18 years	29.2	*	7.38^a^	3.70^b^	<0.000
	Adults 19–64 years	29.9	*	7.45^a^	3.36^b^	<0.000
	Adults 65+ years	26.2	*	5.63^a^	2.44^b^	<0.000
	Adults 65–74 years	26.6	*	5.65^a^	2.44^b^	<0.000
	Adults 75+ years	25.8	*	5.14^a^	2.17^b^	<0.000
Fibre intake (g/d)	Children 1.5–3 years	15.0	2.07^c^	11.60^b^	14.53^a^	<0.000
	Children 4–10 years	20.0	2.56^c^	14.43^b^	17.82^a^	<0.000
	Children 11–18 years	25.0	2.72^c^	17.43^b^	21.52^a^	<0.000
	Adults 19–64 years	30.0	1.94^c^	14.77^b^	18.23^a^	<0.000
	Adults 65+ years	30.0	1.20^c^	9.68^b^	12.20^a^	<0.000
	Adults 65–74 years	30.0	1.23^c^	9.85^b^	12.42^a^	<0.000
	Adults 75+ years	30.0	1.02^c^	8.67^b^	11.00^a^	<0.000
Protein intake (g/d)	Children 1.5–3 years	14.5	50.07^a^	37.79^b^	34.97^b^	<0.000
	Children 4–10 years	24.0	49.74^a^	37.38^b^	34.71^b^	<0.000
	Children 11–18 years	45.9	40.57^a^	30.61^b^	27.89^b^	<0.000
	Adults 19–64 years	50.3	39.21^a^	30.21^b^	26.30^b^	<0.000
	Adults 65+ years	73.7	17.96^a^	14.12^b^	11.78^b^	<0.000
	Adults 65–74 years	73.7	18.28^a^	14.37^b^	11.99^b^	<0.000
	Adults 75+ years	73.7	16.30^a^	12.90^b^	10.57^b^	<0.000
Salt intake (g/d)	Children 1.5–3 years	2.00	18.00^a^	25.00^a^	21.50^a^	0.487
	Children 4–10 years	4.00	15.54^a^	20.82^a^	17.79^a^	0.487
	Children 11–18 years	6.00	14.43^a^	21.15^a^	17.32^a^	0.487
	Adults 19–64 years	6.00	14.15^a^	22.33^a^	16.98^a^	0.487
	Adults 65+ years	6.00	10.32^a^	15.39^a^	11.28^a^	0.487
	Adults 65–74 years	6.00	10.50^a^	15.67^a^	11.48^a^	0.487
	Adults 75+ years	6.00	9.53^a^	14.06^a^	10.11^a^	0.487

^1^Significant differences were declared at *P* < 0.05. Different lower-case letters within a row indicate significant differences between product categories (Tukey’s honest significance test; *P* < 0.05).

### Impact of meat (ME), plant-based (PB) and mycoprotein (MP) products on energy and nutrient intakes and requirements

For Bacon and Ham, there were significant differences between ME, MP and PB products in energy, fat, saturated fat, carbohydrates, sugars, protein, fibre and salt content (*P* < 0.001) (Table 2). The label energy content was highest for ME B&H (*P* < 0.001). ME B&H had 1.6–2.8 times (*P* < 0.001) more declared fat content than PB products. Declared saturated fat for ME B&H was also higher compared with PB and MP (*P* < 0.001). However, the declared carbohydrate content was the lowest for ME B&H (*P* < 0.001), followed by MP, while for PB it was the highest (*P* < 0.001). Additionally, ME B&H had 4.3 times less declared sugar content than PB and less fibre compared with the other two product categories (*P* < 0.001). MP B&H contained intermediate concentrations of sugars. MP B&H protein content was lower than the other two products (*P* < 0.001). ME B&H had more salt content than PB’s and MP’s (*P* < 0.001). These significant differences are then replicated in the individual nutrient intakes from ME B&H, PB B&H, and MP B&H when these were calculated for each consumer age group using the NDNS dataset. Noteworthy implications include a 19-fold increase in fibre from MP in the children 11–18 group (*P* < 0.001; Table 7).

For Burgers and Kebabs, there were significant differences between ME, PB and MP products in energy, fat, saturated fat, carbohydrates, sugars, protein, fibre and salt content (*P* < 0.001) (Table 3). B&K ME had more declared energy, fat, saturated fat, and protein content than PB and MP, while the former one had less declared carbohydrates, fibre and salt content than the other two product categories (*P* < 0.001). In addition, the declared sugar content was 2.5 times (*P* < 0.001) lower for B&K ME than for PBs. These differences are then replicated in the individual nutrient intakes from ΜΕ B&K, PB B&K, and MP B&K when these were calculated for each consumer age group within the NDNS, resulting in changes in a 7.16 times increase in fibre intake from PB compared to ME for children 11–18 years (Table 8).

For chicken turkey and dishes, there were significant differences between ME, PB and MP products in energy, saturated fat, carbohydrates, sugars, protein, fibre and salt content (*P* < 0.001; Table 4). CT&D PB alternatives were higher in declared energy content, followed by ME, and then MP. ME and PB contained more fat (*P* < 0.005) than MP. Declared saturated fat as twice as high (*P* < 0.001) in CT&D ME than in any other product. CT&D ME and MP contained less (*P* < 0.001) declared carbohydrate content than PB products. CT&D PB had the highest sugar, while ME had the lowest. However, declared fibre content was highest (*P* < 0.001) in CT&D MP and lower in CT&D PB, followed by ME. Label protein content was 1.3–1.5 times (*P* < 0.001) highest in ME compared with the other two product categories. CT&D ME had the lowest declared salt content, whilst PB had the highest, and MP contained intermediate concentrations. Individual nutrient intakes from ME CT&D, PB CT&D, and MP CT&D changed when these were calculated for each consumer age group, according to NDNS, resulting in a 1.25-fold rise in PB fat in comparison to ME for adults 19–64 years (Table 9).

For coated chicken and turkey, there were significant differences (*P* < 0.001) between ME, MP and PB products in carbohydrates, sugars, protein, fibre and salt content (Table 5). No significant differences were observed in energy and fat content for CC&T ME, PB and MP. CC&T contained more declared saturated fat than PB products. Label carbohydrates content was higher for PB and MP than for ME (*P* < 0.001). Label sugar content was twice as much for CC&T PB than ME, too (*P* < 0.001). CC&T PB had the highest declared fibre content, while ME had the lowest fibre content and MP showed intermediate values. Declared protein content was 1.2–1.5 times (*P* < 0.001) more for ME compared with MP and PB. However, the declared salt content was less for CC&T ME than for any other product (*P* < 0.001). When looking at the impact on dietary intakes, there was a higher contribution of carbohydrates from MP and PB for all age groups compared to ME (Table 10).

For Sausages, there were significant differences between ME, MP and PB products in energy, fat, saturated fat, carbohydrates, sugars, protein, and fibre content (*P* < 0.001) (Table 6). SAU ME was 1.3 and 1.7 times higher (*P* < 0.001) in declared energy and fat content, respectively, compared with PB. Declared saturated fat content was higher for meat than for MP and PB. SAU ME contained more declared carbohydrate content than PB (*P* < 0.001). Labels on SAU ME suggested these products contained 3.4–3.6 times (*P* < 0.001) less fibre than the other two product categories. However, the declared protein ME was highest for SAU ME. Equally, for label salt content of ME SAU was higher than PB SAU (*P* = 0.013). For label sugar content, there was no difference between ME, PB and MP SAU (*P* > 0.05). These differences affected the individual nutrient intakes from ME SAU, PB SAU, and MP SAU when these were calculated for each consumer age group using the NDNS, resulting in a 1.41-fold decrease of PB protein contribution compared to ME for adults 65+ years (Table 11).

### Substitution of all meat products

Significant differences in energy, fat, saturated fat, carbohydrate, sugar, fibre and protein are observed when ME is substituted with PBMAs across all age groups (*P* < 0.001; Table 12). When ME is replaced by PBMAs, the %EAR would reduce across age groups. Additionally, when replacing ME with PBMAs would result in lower fat, saturated fat and protein contribution of recommended intake across age groups (*P* < 0.001). Noteworthy is the decrease in saturated fat contribution from 4.61% to 6% of Reference Nutrient Intake (RNI) after meat replacement across all age groups. However, in ME substitution with PBMAs, carbohydrate and fibre intake would increase across all age groups (*P* < 0.001). More specifically, the % RNI of fibre would increase significantly between 18.8% and 7.65% across all age groups. No significant differences were detected for salt intake.

## Discussion

Overall, in the present study, PB and MP products contained more carbohydrates, sugars, and fibre, compared with meat products, whilst the energy, protein, fat, and saturated fat content of PB and MP alternatives was significantly lower than that of meat products. Additionally, PB and MP alternatives were significantly more expensive than meat products. Moreover, our analysis highlights the impact of this declared nutrient composition on potential dietary intake across different population age groups.

The energy composition varied significantly between ME, PB and MP alternatives and more specifically, between B&H, B&K, CT&D, and SAU, leading to corresponding changes in energy intake across age groups. For some product categories (B&H, B&K), replacement of meat with PB and MP led to reductions in the energy intake for all age groups, confirming results of other studies^5,26–28^. However, in contrast, PB CT&D had a significantly higher energy content, resulting in increased energy intake for every consumer age group. Similar findings by Zhang et al.^29^ highlighted the variation in the energy profile of products. When ME from the five product groups is combined and substituted with PB and MP products in the NDNS dataset, it results in significant overall differences in energy intake across all age groups. However, this is primarily due to MP, as when ME is substituted with MP, the contribution to EAR is reduced by between ~20% and 30% depending on the age group. However, when ME is substituted with PB, the energy intake did not change. These results indicate that the reduction in energy intake by switching from meat to PBMAs cannot be assumed across the board but depends on the meat category and the corresponding PBMA.

Obesity is prevalent across all age groups in the UK, and causes 31,000 heart and circulatory-related deaths every year^30^. Hence, lower energy consumption from certain PBMAs, particularly MP, replacing specific meat types (e.g., CT&D) can be considered beneficial. But this may not be the case when sausages are substituted by PBMAs. Evidence has shown that switching to PBMA after consuming meat for a period of time is linked to improvements in indicators of cardiovascular disease risk factors, such as lower LDL-cholesterol concentrations and body weight^31^. Additionally, studies support our findings, showing that PBMA, compared to ME, contains higher amounts of fibre, which increases satiety and may support weight maintenance^32^. Potential improvements in lipoprotein profiles, cholesterol, and triglyceride levels were also observed following PBMA consumption compared to ME, which may be linked to this increased fibre content^33^. In accordance with previous studies, Fernández-Rodríguez et al. concluded that PBMA consumption improved total cholesterol, LDL-cholesterol and body weight^34^. Taken together, this research highlights the potential benefits to cardiovascular disease risk when certain meat alternatives are consumed in place of meat.

Children and older people are considered vulnerable population age groups, as their well-being is often fragile and they are more susceptible to malnutrition, while their energy and protein requirements are increased^35–37^. In the 1.5–3-year-old age group, where meat consumption makes up a substantial amount of their daily energy intake (9.52% of their EAR), replacing ME CT&D with PB CT&D would increase their EAR significantly (to +0.67%). Similarly, for adults 75+, moving from ME to MP would decrease %EAR from 5.04% to 3.70% at an age where increased intake of calories is generally required. All the PBMA contained less protein compared to meat. Consequently, this leads to a decreased contribution to RNI of between 3.4% and 15.1% of RNI. These findings comply with a plethora of other studies highlighting a shortfall in protein content of PBMA^26,27,38,39^. This is of particular relevance to children and older adults, where an adequate intake of high-quality protein plays a pivotal role in growth and health^40^. Proteins contribute to the development, maintenance and repair of children’s bodies, while older adults are at risk of sarcopenia due to the progressive loss of muscular mass, strength, and function that occurs with aging^40^. Guidelines for dietary protein recommend 0.8 g/kg body weight/day for healthy adults, 1–1.2 g/kg body weight/day for older adults or 1.2–1.5 g/d body weight/day for older people who are malnourished or at risk of malnutrition to sustain their muscle protein synthesis^36^. From the NDNS dataset, adults over 75 years of age obtain 18.9% of their protein requirements from meat sources. With the substitution of PB and MP, only 12.9% and 10.6% of their protein requirements would be met through these sources. Considering that most PB sources lack essential amino acids and fail to cover the protein demands of older people, PB meat alternatives might not be a suitable protein source for older people^41^. More specifically, 80–95% of plant proteins are usually digested in the human gastrointestinal tract (GIT), instead of 95–100% of meat proteins. Hence, with the same overall total protein intake, people may receive less protein from plant-based foods than from animal-based ones^42^. Additionally, plant-based proteins lack a complete essential amino acids profile, and the high level of processing in PBMA can hinder amino acid absorption by disrupting plant cell structures^42^. A potential solution to this issue could involve combining proteins from various plant-based sources and improving processing methods to enhance the nutritional quality of these products^39,42^.

There were significant differences in the total fat and saturated fat content between PBMA and meat. Across all meat categories, both PB and MP had lower fat and saturated fat content than ME, with the exception of PB CT&T and CT&D, which had a similar total fat to ME but still had a lower saturated fat. This has consequent impacts on contributions to intakes across age groups. These findings agree with other studies indicating that the majority of PBMAs have a lower fat and saturated fat content^27,29,43^. However, findings from McClements and McClements^42^. demonstrated that PB nuggets contained less fat and saturated fat than chicken meat. This contradicts our results, which found that PB CC&T and CT&D were similar in total fat content to that of ME, and work by Lindberg et al. that found that ME chicken products had significantly lower saturated fat^44^. This highlights the differences between countries and studies depending on the products available in different markets. Daily intake of saturated fatty acids exceeds recommendations in the UK for adults aged 65 years and over, which is linked to elevated LDL-cholesterol levels and consequently risk of development of CVD^45,46^. As such, replacement of saturated fat with unsaturated or polyunsaturated fat, plant-based protein and complex carbohydrates could have a positive impact on minimising the risk for CVD development^47^. Substituting ME with PBMA resulted in a reduction in dietary saturated fat intake, which accounted for 2.58% for PB and 3.04% for MP. The literature suggests a reduction or replacement of 5–6% of daily energy from saturated fat with other nutrients in order to see an improvement in blood lipids^30,48^, meaning these differences are likely to have a negligible impact on human health.

It is noteworthy that all PBMAs contain higher amounts of carbohydrates and fibre, with PB products also containing more sugar. However, there was some variation with product type PB CT&D containing more carbohydrates, which resulted in three times the contribution of carbohydrates to intake recommendations across age groups. A higher amount of carbohydrates in ME CC&T products is owed to the existence of breadcrumb^42^; however, this was still lower than PB and ME options in this category. The current study confirmed the higher sugar content of PBMA observed across a number of studies^28,42,43^; however, this was not found so consistently or overall for MP products. Added sugars in refined food products have been correlated with cardiovascular, metabolic and mental disorders^49,50^. However, since meat does not contain free sugar, its contribution to intake guidelines was not completed in this analysis.

Significant increases in fibre intake were observed following meat substitution with PBMA. ME substitution with MP product resulted in a 7–10 times greater intake of fibre across all age groups. Moreover, products from MP contained the most fibre in all food categories other than B&K, in which PB ranked first. Dietary fibre has a significant impact on lowering serum total and low-density lipoprotein (LDL) cholesterol concentrations, and the glycaemic index of foods, both in adults and children. Therefore, higher consumption of fibre can decrease the prevalence and risk of cardiovascular disease, type 2 diabetes, breast and colorectal cancer^51,52^. Since most people in the UK fail to obtain a sufficient intake of fibre from their diets, it is critical to increase their intake. UK guidelines propose intakes of between 15 and 25 g/d for children above 2 years of age, for adults, the recommended intake of fibre is 30 g/d^53^. Moving from ME to either PB or MP across the diet could have a significant impact on achieving the recommended intake. For example, in the 11–18 years old age group, meat provided 2.72% of the RNI of fibre, where moving to PB increased this to 17.43% of RNI and to MP to 21.53% of RNI. Consequently, both PB and MP versions with a high fibre content would be beneficial for consumers’ fibre intake. Similar findings highlighting the high fibre content of PBMA have been consistently reported across previous studies^24,26,27,29,43,44^. As such, PBMA can be a beneficial dietary choice for consumers looking to increase fibre in their diet.

From the results, the salt content did not differ significantly among products; however, it was significantly different between all types of product categories. For B&H and SAU, the salt content in ME was highest. However, for B&K and CC&T, the MP and PB had the highest salt content, while the PB CT&D contained the highest amounts of salt. In the 1.5–3 years old age group, substitution of ME with PB CT&D would increase salt consumption by ~8% as a percentage of requirements, while substituting ME with MP would increase salt intake by 3.5% of requirements. Mixed results were presented from other studies with majority, but not all^21^, showing that PB products had a higher salt content than meat^24,26–28,43,44^. Foods are considered to be high in salt if they contain above 1.5 g/100 g of salt^54^. Mean PB salt content, which was 1.44 g/100 g (*P* < 0.01) would be considered moderate in salt content, however the highest of the categories was ME B&H with 3.32 g/100 g. Excessive dietary sodium intake could lead to increased levels of blood pressure, incidence of hypertension, but also the morbidity and mortality due CVD^55,56^; however, on average, in the current UK PBMA product market, a high salt content does not appear to be an issue.

In agreement with previous research^26,27,39,43^, our results revealed that all PBMAs were more expensive than all meat products and might not be affordable to a typical UK household. Prices of PBMA can be 38-73% more expensive than their meat equivalents per 100 g, and younger age groups tend to have more limited spending capacity^22^. This has been attributed by manufacturers to the lower sales volumes and therefore small production runs, as well as stricter technical precautions associated with vegan products^57^. Depending on the foods consumed, regular consumption of PBMA can have a high monetary cost to consumers.

The nutritional composition of PBMA varies considerably across product categories due to different ingredients and is significantly different from ME. Plant-based diets have been found to be healthier than consuming meat; however, this may not be the case when large amounts of PBMA are substituted, as most of these foods are considered ultra-processed, which is linked to harmful health effects^38^. The addition of artificial colourings, excessive sugars, and salt (in some products) in an attempt to enhance and mimic meat’s taste, texture, smell and appearance could lead to obesity and other comorbidities^25,58^. PBMA are also lower in many micronutrients of interest, such as iron, vitamin B12, zinc and omega-3-fatty acids, and the digestibility of plant foods is generally lower compared to foods derived from animals, reducing absorption of micronutrients^42^. It is, therefore, critical to recommend cautiousness in daily consumption of PBMA.

In contrast, however, plant-based alternatives have been demonstrated to have a lower Nutri-Score due primarily to the higher quantity of fibre in the products^28^. The health benefits of fibre have already been mentioned, but most importantly, a healthy gut microbiome is strongly associated with foods rich in fibre^42^. Thus, a healthier microbiome was observed when participants consumed five plant-based meat alternative meals in a week rather than meat meals^59^. Another asset of plant-based alternatives is that they have a small environmental impact with lower greenhouse gas emissions. There is a significant difference in CO_2_ emissions between meat and plant-based foods, with the former having the most^26^ and consumption of plant-based foods may play a significant role in transitioning towards sustainable diets.

The current study provides findings from the largest database of plant-based meat alternatives and meat comparison products recorded in the UK; however, it is not without its limitations. This study was conducted between July and December 2021; thus, prices, along with the existence of products, might have changed. Due to the abundance of data already reported in this paper, NDNS meat categories with less PB or MP equivalent products were excluded from this study, including ‘Beef, veal and dishes,’ ‘Lamb and dishes,’ ‘Pork and dishes,’ and ‘Liver and dishes’, and fish products. Moreover, meat products were collected only from two supermarkets, as there is a substantial overlap in the types of meat products available across different supermarkets. However, we recognise there may be variations in food composition based on country of origin, type of meat cut and animal species, and across brands. In the present study, only product label data were included, and micronutrients were not examined, as it is not mandatory to declare micronutrient content on-pack in the UK unless the product has been fortified or makes a micronutrient-related claim. Certain models of nutrient profiling showed that micronutrient content, including zinc, iron and B12, changed when substitution is made^5,24,25^. Additionally, the annual sales value of plant-based meat substitutes in the UK has seen a decrease by 2.8% between 2022 and 2023, indicating a decline in consumer engagement with these products; as such, the implications for this work may be reduced if this trend continues^9^.

In conclusion, PBMA cost almost twice as much as ME products, have lower energy, protein, fat, and saturated fat content than ME. On the other hand, they are rich in carbohydrates and fibre. Differences did exist between product categories and between PB and MP products; therefore, it is important to pay significant attention to product labels and nutritional requirements in different age groups when substitutions are made.

Future research should consider the impact of substituting ME products with PBMA on a wider range of nutrients, including micronutrients and the impact on population health outcomes and environmental sustainability. In particular, additional studies are needed to assess the effects of PBMA consumption on established health biomarkers, blood pressure, and body composition. Concurrently, comprehensive life cycle assessments should quantify the environmental impacts of PBMA production and consumption, including greenhouse gas emissions, land use, and water footprint. Such multidisciplinary research is essential to determine whether PBMA offers meaningful advantages over traditional meat products, providing a more holistic understanding of the potential benefits and trade-offs associated with a dietary shift from animal-based to plant-based protein sources.

## Methods

The data for PBMA and meat products was collected from the website of six major supermarkets in the UK, including Morrisons, Tesco, Sainsbury’s, Waitrose, Ocado, Asda and Aldi (collectively covering ~73% of grocery market share in 2020). The products were selected by reviewing the ‘Meat and Poultry’ category for meat products, and the ‘Vegan’ and ‘Meat free’ categories for PBMA products on supermarket websites. Information on meat products was collected from two supermarkets, Tesco and Morrisons (covering ~37% of the grocery market share in 2020), due to the vast number of similar meat products between supermarkets. Data was collected from July 2021 to September 2021. Both the retailer’s and manufacturer’s websites were checked to ensure all information was correct.

A database was created which included the following information: Name of the product; production system (conventional, organic); storage conditions (refrigerated, frozen); brand; price of the product and price per 100 g (£/100 g); product description; type of food in the label and type of food classification according to NDNS^11^; web link; package size (g); serving size (g); serving size description. The nutritional data gathered included energy (kcal/100 g), fat (g/100 g), saturated fat (g/100 g), monounsaturated fat (g/100 g) and polyunsaturated fat (g/100 g), carbohydrates (g/100 g), sugars (g/100 g), fibre (g/100 g), protein (g/100 g) and salt (g/100 g).

### Exclusion criteria

Ready meals were excluded from this study. Where there were two identical products in different quantities, the larger size product was included, and the smaller one was omitted from the database. Vitamins and minerals were not recorded because limited information on them was provided on nutrition labels.

### Data categorisation

Meat protein substitute products can be difficult to classify into specific categories. Hoek et al.^60^ categorised meat substitutes into kinds of animal source (beef, pork, and chicken), into the food characteristic product form (sausages, burgers, slices, balls, minced meat, satay, fungi, legumes, and snacks). On the other hand, PBMA could be grouped according to their primary ingredients into three or four categories: PBMA derived from plant sources, such as legumes, vegetables and nuts; fungi-based meat alternative products made from microorganisms; insect-based meat analogue products using insect protein sources and culture-based meat constructed from animal cells in laboratories^61,62^.

In the present study, to make comparisons across the UK diet, foods were categorised into food type as they are classified in the NDNS dataset: B&H, B&K, CT&D, CC&T, and SAU. We considered each product within our PBMA and MP dataset from the retail survey, and we matched these products to the corresponding categories in the National Diet and Nutrition Survey (NDNS) dataset. As an example, meat-free sausages, as described by the manufacturers, were classified under the SAU category in the NDNS dataset. Details of the disaggregated foods available in the NDNS dataset are available on the UK data service webpage^63^, and NDNS data were available from Public Health England^11^. Meat categories also present in the NDNS were ‘Beef, veal and dishes’, ‘Lamb and dishes’, ‘Pork and dishes’ and ‘Liver and dishes’, however, these were not analysed as there were no PBMA or MP products that aimed to match these categories. Foods were then further grouped into three sub-categories: ME, PB, for all kinds of plant-based products, and MP for products produced from a naturally occurring fungus *Fusarium venenatum*. Tofu on its own (not incorporated into a product) was also excluded from our analysis since it is not a direct PBMA of any of the meat products within these categories.

### Nutritional intake from meat, plant-based and mycoprotein products

Average daily intake of bacon and ham, burger and kebabs, chicken turkey and dishes, coated chicken and turkey, and sausages was found in the NDNS data years 9–11 (conducted in 2016/17–2018/19)^11^. Intake data in the NDNS is based on a 4-day estimated diet diary and is divided into different age categories (1.5–3 years; 4–10 years; 11–18 years; 19–64 years; 65 + years; 65–74 years; 75 + years), and these categories were used throughout our analysis. Intakes for bacon and ham, burger and kebabs, chicken turkey and dishes, coated chicken and turkey, and sausages product categories from NDNS data were given as a percentage of total energy intake per age group. To convert the NDNS intake data from a percentage of energy intake to absolute intake in grams, the means of energy of all categories of meat products, as averaged from the data collected in the present study, were used. Intake for all meat product categories, as presented in NDNS^11^, was multiplied by the means of the nutrients (fat, saturates, carbohydrates, sugars, fibre, protein, and salt) individually to calculate the specific intake of nutrients from every meat and PBMA product category and demographic group (age, sex). This allowed the exploration of a scenario whereby meat in the diet was entirely replaced by the corresponding plant-based or mycoprotein products on a gram-for-gram basis, and the quantification of the impact on the consumer's energy and nutrient intakes when meat is replaced with PBMA. Following this, the calculated energy and nutrient intakes from the consumption of the different product types were compared to the UK dietary reference values (DRV). Estimated average requirements (EAR) for energy intake were derived from the average of male and female intake, and for middle-aged groups for children (e.g., 2-year-old EAR data was selected in the 1.5-3-year age group; 7-year-old EAR data was selected in children aged 4–10 years)^64^. For fat, 35% of daily food energy was applied and no more than 10% of daily food energy for saturated fat for populations aged 5 years and above. Protein was based on the RNI for adults and children, and for older adults, the higher recommendation by the European Society for Clinical Nutrition and Metabolism (ESPEN) of 1.1 g/kg was applied^36^. DRVs for carbohydrate amounted to 50% of total daily food energy, as outlined in the Scientific Advisory Committee on Nutrition report on Carbohydrate, with recommendations for fibre coming from the same report. Salt was set at the target salt intakes for adults and children^65^. Sugar in meat is not classified as free sugar; consequently, this comparison was not carried out.

### Data analysis

The difference between ME and PB and ME products, within the different type of products, as well as the impact of substituting ME with PB and MP on nutrient intake of different age groups, was assessed by conducting an analysis of variance (ANOVA), using a linear regression model, using main ingredient, type of product, and their interaction as fixed effects in Minitab 21 Statistical Software^66^. When the effect of the fixed factors or their interaction was statistically significant, pairwise comparisons of means (*P* < 0.05) were performed using Tukey’s Honest Significance test to identify significant differences between the treatment means.

In order to analyse the comparisons between main ingredients within each type of product in further detail, one database for each type of product was created (B&H, B&K, CT&D, CC&T, SAU). Each of the five databases was then analysed by linear models using the main ingredient as a fixed factor. When the effect of the fixed factor was statistically significant, pairwise comparisons of means (*P* < 0.05) were performed using Tukey’s honest significance test.

## Data Availability

The datasets generated and/or analysed during the current study are not publicly available but are available from the corresponding author on reasonable request.
